# Application of Near-Infrared Spectroscopy in Moisture Detection of Carrot Slices During Freeze-Drying

**DOI:** 10.3390/foods15071256

**Published:** 2026-04-07

**Authors:** Pengtao Wang, Meng Sun, Hongwen Xu, Moran Zhang, Rong Liu, Yunfei Xie, Jun Cheng

**Affiliations:** 1 Wuxi Rilian Vision Technology Co., Ltd., Wuxi 214028, China; wangpt@unicomp.cn; 2 School of Food Science and Technology, Jiangnan University, Wuxi 214122, China; 6220112072@stu.jiangnan.edu.cn (M.S.); 7220112102@stu.jiangnan.edu.cn (H.X.); 7250112074@stu.jiangnan.edu.cn (M.Z.); 7250112036@stu.jiangnan.edu.cn (R.L.); xieyunfei@jiangnan.edu.cn (Y.X.)

**Keywords:** freeze-dried carrots, moisture content, near-infrared spectroscopy

## Abstract

This study explored the feasibility of near-infrared (NIR) spectroscopy for detecting total water, free water and bound water in carrot slices during freeze-drying, with low-field nuclear magnetic resonance (LF-NMR) characterizing water state distribution and oven-drying determining moisture content (MC). NIR spectra (10,000–4000 cm^−1^) were processed via optimized sample partitioning, preprocessing and feature extraction; partial least squares regression (PLSR), support vector regression (SVR), back-propagation artificial neural network (BPANN), extreme gradient boosting (XGBoost) and particle swarm optimization–random forest (PSO-RF) models were established and evaluated. Results showed that SVR and BPANN performed robustly, with CARS being the optimal feature extraction method. The full-moisture system achieved high total/free water prediction accuracy ($R_{p}^{2}$ = 0.9902/0.9740), while the low-moisture system improved bound water prediction ($R_{p}^{2}$ = 0.9709). The established NIR models exhibited excellent fitting and generalization ability, enabling rapid and non-destructive quantitative prediction of moisture content during carrot freeze-drying.

## 1. Introduction

Near-infrared (NIR) spectroscopy is an analytical technique based on molecular vibrational and rotational absorption characteristics. Owing to its mature instrumentation, operational simplicity, rapid response, and non-destructive nature, NIR spectroscopy has been widely applied in food and agricultural product analysis, particularly for moisture determination [1]. In the NIR spectral region (900–2500 nm, corresponding to 10,000–4000 cm^−1^), hydrogen-containing functional groups such as O–H, N–H, and C–H undergo overtone and combination vibrations, resulting in characteristic absorption features at specific wavelengths [2]. Through transmission, reflection, or diffuse reflection interactions between NIR radiation and the sample, spectral fingerprints composed of absorption band positions, intensities, and shapes are generated, providing comprehensive information on the chemical composition and physical structure of the sample [3]. Water molecules exhibit distinct absorption bands (Table 1), and the absorption intensity correlates strongly with moisture content (MC). Therefore, integrating NIR spectroscopy with chemometric or machine learning approaches enables rapid and accurate moisture prediction [4].

Over the past decades, NIR spectroscopy combined with multivariate modeling techniques has been extensively investigated for the determination of residual moisture in dried products, serving as an effective alternative to conventional time-consuming and destructive methods such as Karl Fischer titration [5]. In the field of fruit and vegetable drying, NIR spectroscopy has also demonstrated excellent applicability and reliability. Jin et al. [6] employed NIR spectroscopy to achieve real-time assessment of moisture content in apple slices during hot-air-assisted radio frequency drying, where the back-propagation artificial neural network (BPANN) model achieved the highest prediction accuracy ($R_{p}^{2}$ = 0.976). Marinoni et al. [7] utilized a portable NIR spectrometer to monitor physicochemical changes in melon slices during solar drying, and the developed partial least squares regression (PLSR) models exhibited strong predictive performance for moisture content (R_CV^2^ = 0.99) and water activity (R_CV^2^ = 0.97). Zhang et al. [8] integrated NIR spectroscopy with machine learning algorithms to develop a non-destructive and efficient method for real-time moisture monitoring of dried *Porphyra yezoensis*, in which the extreme gradient boosting (XGBoost) model achieved optimal prediction performance (R^2^ = 0.979). These studies collectively demonstrate the strong potential of NIR spectroscopy for monitoring moisture dynamics during the drying and storage of agricultural products.

With the rapid advancement of machine learning techniques, the application of NIR spectroscopy for precise moisture prediction in agricultural products has been further expanded. Zou et al. [9] developed an XGBoost–CNN–TS–EN model based on NIR spectra for predicting maize moisture content, achieving high prediction accuracy and strong generalization capability. Widyaningrum et al. [10] investigated the potential of portable NIR spectroscopy for moisture determination in vanilla, where a random forest model effectively exploited spectral information to achieve excellent prediction performance. Parrenin et al. [11] established NIR-based machine learning models for quantitative analysis of wheat moisture content, obtaining an R_CV^2^ value of 0.90 with good prediction stability. Ordoñez et al. [12] evaluated the feasibility of NIR spectroscopy for rapid and non-destructive prediction of moisture content in coffee beans during drying, and the developed principal component regression (PCR) model exhibited excellent performance (R^2^ > 98%, RMSE < 3.4%). Nevertheless, existing studies have predominantly focused on the prediction of total moisture content, while quantitative modeling of different water states, such as free water and bound water, during freeze-drying remains insufficiently explored.

Therefore, the objective of this study is to systematically investigate the feasibility of NIR spectroscopy for moisture detection in carrot slices at different stages of the freeze-drying process and to evaluate its potential for predicting changes in total water, free water, and bound water contents. NIR spectra of samples with varying moisture levels were collected, while LF-NMR was employed to characterize water state distributions. Total moisture content was determined using the oven-drying method, and the contents of free and bound water were subsequently calculated. Based on the established relationships between spectral data and moisture parameters, multiple strategies for sample partitioning, spectral preprocessing, feature extraction, and model construction were systematically compared. Prediction models for total water, free water, and bound water were developed for both the entire freeze-drying process (MC < 100%) and the late drying stage (MC < 20%), and their predictive performance and applicability were comprehensively evaluated.

## 2. Materials and Methods

### 2.1. Raw Materials and Sample Preparation

Fresh Sanhong carrots (origin: Weifang, Shandong, China) were purchased from a local supermarket (Dingdong Maicai, Wuxi, China). The initial moisture content of the carrots was 91.62 ± 0.82% (wet basis, w.b.); the initial moisture content of fresh carrots was determined using the oven-drying method (105 °C, 24 h) as described in Section 2.3.3. Carrots with uniform size, intact appearance, and no mechanical damage were selected. After removing surface impurities, the samples were sliced into circular discs with a diameter of approximately 30 mm and a thickness of 3 mm. The slices were evenly arranged on freeze-drying trays with a loading density of 0.80 kg/m^2^ to ensure uniform heat and mass transfer.

The freeze-drying parameters are presented in Table 2. The process consisted of a pre-freezing stage (Stage 1), a vacuum sublimation stage (Stage 2), and a constant-temperature drying stage (Stage 9). Freeze-drying formally commenced from Stage 2, and samples were collected at 4 h intervals throughout the process until completion. The final moisture content of the freeze-dried samples complied with the requirement of GH/T 1326–2021 [13] for freeze-dried fruits and vegetables (<0.08 g/g, w.b.).

### 2.2. Instruments

The main instruments used in this study included an Antaris II Fourier transform near-infrared spectrometer (Thermo Fisher Scientific, Waltham, MA, USA), a MicroMR20-030V-I low-field nuclear magnetic resonance (LF-NMR) analyzer (Niumag, Shanghai, China), a YHT 103251 digital thickness gauge (Yuanhengtong, Shenzhen, China), an AB104-N analytical balance (Mettler Toledo, Shanghai, China), a SCIENTZ-10YD/A vacuum freeze dryer (Ningbo Scientz Biotechnology Co., Ltd., Ningbo, China), and a DHG-91013SA electric blast drying oven (Shanghai Sanfa Scientific Instrument Co., Ltd., Shanghai, China).

### 2.3. Experimental Methods

#### 2.3.1. FT-NIR Spectral Acquisition

Fourier transform near-infrared (FT-NIR) spectra were collected over a wavenumber range of 10,000–4000 cm^−1^. Samples were placed in a rotating quartz cup and measured using an integrating sphere in diffuse reflectance mode. The spectral resolution was set to 8 cm^−1^, and 64 scans were accumulated for both background and sample measurements to improve the signal-to-noise ratio. Each sample was scanned independently three times, and the average spectrum was used for subsequent analysis.

#### 2.3.2. Low-Field Nuclear Magnetic Resonance (LF-NMR) Measurements

Samples were placed into NMR tubes for the analysis of water state distribution. After calibration using the free induction decay (FID) sequence, proton transverse relaxation signals were acquired using the Carr–Purcell–Meiboom–Gill (CPMG) pulse sequence. The relaxation decay signals were processed using an inversion algorithm to obtain T_2_ relaxation spectra, which were used to characterize different water states. Each sample was measured in triplicate, and the mean value was reported. In the T_2_ relaxation spectra, A_21_, A_22_, A_23_, and A_24_ represent the peak areas corresponding to strongly bound water, weakly bound water, immobilized water, and free water, respectively. These peak areas are proportional to the relative content of each water state and are expressed as dimensionless values.

#### 2.3.3. Determination of Moisture Content During Freeze-Drying

Moisture content was determined according to the direct oven-drying method (Method I) specified in GB 5009.3-2016 [14]. Samples were dried at 105 °C for 24 h until constant weight was achieved. Moisture content was calculated using Equation (1), where *Y* represents the moisture content of the sample at time t (w.b.), m_t_ is the fresh mass at time t (g), and m_0_ is the dry mass after oven-drying (g).

(1) $$Y=\frac{m_{t}-m_{0}}{m_{t}}\times 100\%$$

#### 2.3.4. NIR Spectral Data Processing and Quantitative Model Development

FT-NIR spectral preprocessing, feature extraction, and model development were conducted using Matlab 2022a (MathWorks, Natick, MA, USA), Unscrambler X 10.4 (CAMO Software, Oslo, Norway), and Origin 2021 (OriginLab, Northampton, MA, USA).

1. Sample set Partitioning

The Kennard–Stone (KS) and Sample set Partitioning based on joint X–Y distances (SPXY) algorithms were applied to divide samples into calibration and prediction sets at a ratio of 3:1.

2. Spectral preprocessing

To reduce noise and eliminate non-informative variations, spectral preprocessing methods including moving average (MA), Savitzky–Golay smoothing (SG), standard normal variate (SNV), normalization (Nor), multiplicative scatter correction (MSC), orthogonal signal correction (OSC), first derivative (FD), and second derivative (SD) were evaluated.

3. Feature variable selection

Competitive adaptive reweighted sampling (CARS), successive projections algorithm (SPA), and uninformative variable elimination (UVE) were employed to select characteristic variables and reduce spectral redundancy.

4. Model development and evaluation

PLSR, support vector regression (SVR), BPANN, XGBoost, and particle swarm optimization–random forest (PSO-RF) models were developed. Model performance was evaluated using the coefficients of determination for calibration and prediction (R_C^2^ and R_P^2^), root mean square errors of calibration and prediction (RMSEC and RMSEP), and residual predictive deviation (RPD).

#### 2.3.5. Sample Characterization

1. Total number of samples analyzed

A total of 300 carrot slices were analyzed throughout the freeze-drying process. For each of the 10 sampling time points (0, 4, 8, 12, 16, 20, 24, 28, 32, 36 h), 30 slices were collected. For each slice, FT-NIR spectra were acquired in triplicate, and LF-NMR measurements were performed in triplicate. Oven-drying moisture content was determined in triplicate for each slice.

2. Number of experimental replicates

All experiments were performed with three independent replicates (n = 3) for each sample.

3. Sample mass

The average fresh mass of carrot slices before freeze-drying was 2.85 ± 0.12 g (n = 300). The average dry mass after oven-drying was 0.24 ± 0.02 g.

## 3. Results

### 3.1. Moisture Dynamics and State Distribution During Freeze-Drying of Carrot Slices

**Figure 1:**
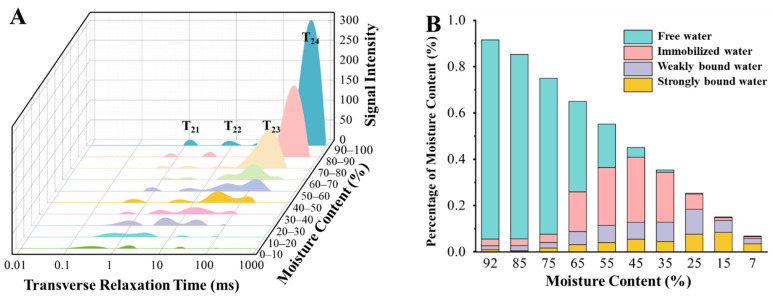
Water status of carrots under different moisture gradients. (**A**) T_2_ relaxation spectra of carrot slices at different moisture contents during freeze-drying. The labels T_21_, T_22_, T_23_, and T_24_ indicate strongly bound water, weakly bound water, immobilized water, and free water, respectively. (**B**) Relative proportions of different water states as a function of moisture content during freeze-drying.

The T_2_ relaxation spectra shown in Figure 1A clearly illustrate the dynamic moisture removal behavior and water state evolution of carrot slices during freeze-drying. At the initial drying stage, the T_24_ peak corresponding to free water predominated, and its signal intensity decreased rapidly as freeze-drying progressed, indicating the preferential removal of free water. In contrast, the peak associated with immobilized water (T_23_) exhibited a more gradual decline and mainly decreased during the middle and late stages of freeze-drying, suggesting that the removal of immobilized water requires higher drying driving forces and longer processing times. The signal intensity of weakly bound water (T_22_) showed relatively minor fluctuations, while the strongly bound water peak (T_21_) remained nearly unchanged throughout the entire process, demonstrating the high structural stability of bound water, which is the most difficult fraction to remove during freeze-drying.

The variations in the proportion of different water states presented in Figure 1B, together with the peak area data summarized in Table 3, further confirmed the observed moisture removal patterns. As freeze-drying proceeded and the total moisture content continuously decreased, free water was preferentially removed, and its proportion was gradually replaced by immobilized and bound water fractions, which is consistent with the findings reported by Sun et al. [15].

From a stage-wise perspective, free water decreased rapidly during the early phase of freeze-drying due to its weak interaction with the solid matrix. When the moisture content of the samples decreased to approximately 30–40%, free water was almost completely removed. The loss of free water induced a redistribution of internal moisture, exposing additional hydrophilic binding sites on macromolecular surfaces and resulting in a temporary increase in immobilized water content [16]. With the continuation of freeze-drying, immobilized water became increasingly affected by capillary effects, molecular diffusion, and sublimation-driven mass transfer, leading to a gradual decrease during the middle and late stages. When the moisture content dropped to 0–10%, immobilized water was nearly completely eliminated.

In comparison, weakly and strongly bound water exhibited much smaller variations throughout the freeze-drying process. During the early stage, their contents slightly decreased due to the reduction in environmental moisture. In the middle stage, cellular concentration and the increase in available binding sites led to a temporary increase in these water fractions. Only in the final stage of drying did their contents gradually decline with further dehydration [17]. Notably, at the end of freeze-drying, the highly concentrated tissue structure enabled residual water molecules to be more tightly adsorbed by polar macromolecules, resulting in slightly higher relative contents of weakly and strongly bound water compared to the initial stage.

Overall, moisture removal during the freeze-drying of carrot slices exhibited pronounced selectivity and stage-dependent behavior, with the preferential removal sequence following the order free water > immobilized water > weakly bound water > strongly bound water.

### 3.2. Near-Infrared Spectral Characteristics During Freeze-Drying of Carrot Slices

**Figure 2:**
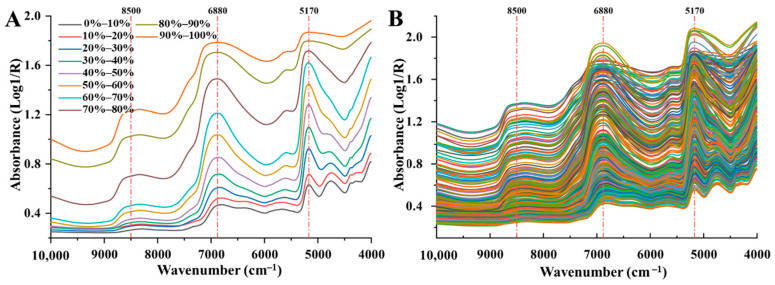
NIR spectra. (**A**) NIR signals of samples with different MC; (**B**) all NIR spectra.

The near-infrared (NIR) spectra of freeze-dried carrot samples at different moisture contents are presented in Figure 2. Overall, a gradual decrease in absorbance across the entire wavenumber range was observed as the freeze-drying process progressed and moisture content decreased. The most pronounced absorbance variations occurred at approximately 8500 cm^−1^, 6880 cm^−1^, and 5170 cm^−1^. These characteristic bands are closely associated with O–H vibrational modes of water molecules: the absorption band at around 8500 cm^−1^ is attributed to the second overtone of O–H stretching vibration [18], the band near 6880 cm^−1^ mainly arises from the first overtone of O–H stretching vibration [4], and the absorption at 5170 cm^−1^ corresponds to a combination of O–H stretching and bending vibrations [19]. These results clearly indicate that variations in moisture content exert a direct and significant influence on the NIR spectral response of freeze-dried carrot samples.

A closer examination of spectral variations across different moisture ranges reveals that when the moisture content was relatively high (40–90%), absorbance decreased markedly with moisture loss, and substantial spectral differences were observed among samples. At this stage, water predominantly existed as free water and immobilized water, both of which exhibit relatively weak interactions either among water molecules or with the solid matrix. Consequently, these water fractions show strong NIR absorption and play a dominant role in shaping the spectral response. In contrast, when the moisture content decreased below 40%, changes in absorbance became less pronounced and spectral differences among samples diminished. Under these conditions, water was mainly present in the form of bound water. Due to the formation of stable hydrogen-bonding networks between bound water and polar macromolecules, molecular mobility is restricted, resulting in a weaker but more stable contribution to NIR absorption.

Taken together, these findings demonstrate that NIR spectroscopy is highly sensitive to moisture variations during the freeze-drying of carrot slices, particularly in the high-moisture stage dominated by free and immobilized water. This pronounced spectral response provides a solid basis for the rapid and non-destructive quantification of total moisture content during freeze-drying, and further supports the feasibility of distinguishing different water states and developing stage-specific moisture prediction models.

### 3.3. Near-Infrared Spectral Data Processing

#### 3.3.1. NIR Spectral Sample Partitioning

Based on NIR spectra, quantitative models were developed for different drying stages and water types to enable accurate moisture prediction during freeze-drying. Six models were established, covering the entire freeze-drying process (overall moisture, MC < 100%) and the late drying stage (low-moisture, MC < 20%), including total water, free water, and bound water. The overall moisture models were designed to capture the global moisture removal trend, whereas the low-moisture models focused on subtle changes at the final drying stage to improve endpoint discrimination.

The Kennard–Stone (KS) and Sample set Partitioning based on joint X–Y distances (SPXY) algorithms were applied to divide samples for each model, and the results are summarized in Supplementary Table S1. The calibration and prediction sets exhibited good coverage across the target moisture ranges, indicating that both methods ensured representative and well-balanced sample distributions, which is essential for robust spectral learning.

It should be noted that the target range of the low-moisture free water model was relatively narrow (0–0.86%). Such a limited moisture variation may reduce spectral discrimination and consequently constrain model generalization and predictive performance [20]. In contrast, the remaining models covered wider moisture ranges, providing a more robust data basis for model development and validation.

#### 3.3.2. NIR Spectral Preprocessing

**Figure 3:**
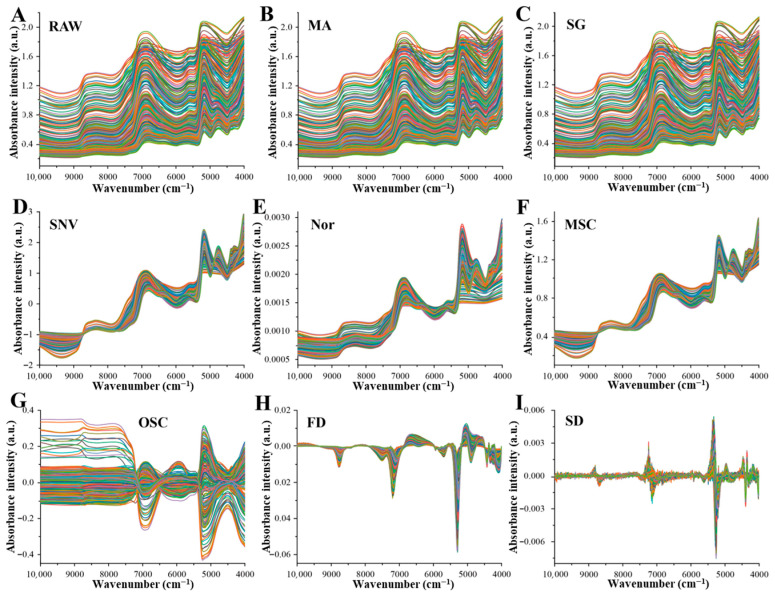
Preprocessing of NIR spectra under full-moisture conditions. (**A**) Raw spectra, (**B**) moving average (MA), (**C**) Savitzky–Golay smoothing (SG), (**D**) standard normal variate (SNV), (**E**) normalization (Nor), (**F**) multiplicative scatter correction (MSC), (**G**) orthogonal signal correction (OSC), (**H**) first derivative (FD), (**I**) second derivative (SD).

**Figure 4:**
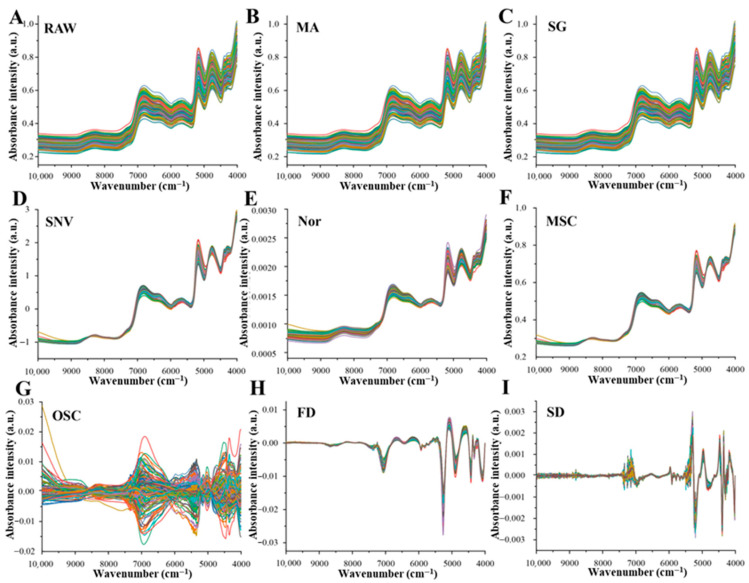
Preprocessing of NIR spectra under low-moisture conditions. (**A**) Raw spectra, (**B**) moving average (MA), (**C**) Savitzky–Golay smoothing (SG), (**D**) standard normal variate (SNV), (**E**) normalization (Nor), (**F**) multiplicative scatter correction (MSC), (**G**) orthogonal signal correction (OSC), (**H**) first derivative (FD), (**I**) second derivative (SD).

Raw NIR spectra are often affected by baseline drift, light scattering, and instrumental and environmental noise, which may obscure spectral information directly related to water content. To enhance model robustness and predictive performance, eight spectral preprocessing methods were applied after sample partitioning, and SVR models were subsequently established. As shown in Supplementary Tables S2 and S3 and Figure 3 and Figure 4, both sample partitioning strategies and preprocessing methods exerted pronounced effects on model performance.

Regarding sample partitioning, the SPXY algorithm is suitable for datasets with strong correlations between spectral variables and target properties [21], whereas the KS algorithm emphasizes uniform coverage of the spectral space and is more appropriate for datasets with large spectral variability [22]. The modeling results indicate that both approaches can yield stable calibration and prediction performance, depending on their compatibility with specific moisture systems and preprocessing strategies.

In the full-moisture system, the total water exhibited broad absorption bands with strong signal intensity, making it relatively insensitive to scattering interference. As shown in Supplementary Table S2, KS+MSC achieved the best performance ($R_{p}^{2}$ = 0.9883), effectively reducing scattering effects in the high-wavenumber region (Figure 3F) [23].

In contrast, free water is characterized by narrower and sharper absorption peaks, making its spectral signal more susceptible to coupled interference from scattering and baseline fluctuations. Among all preprocessing methods, only first derivative (FD) preprocessing significantly improved model performance, with the KS+FD combination yielding the highest prediction accuracy ($R_{p}^{2}$ = 0.9620). Comparison of Figure 3A and Figure 4H shows that FD enhanced local spectral features in the 6000–5000 cm^−1^ region, which was critical for capturing weak free water absorptions.

For the full-moisture bound water model, spectral overlap between bound water and other components posed a major challenge, as bound water absorptions often appeared as shoulders or overlapped bands. As indicated in Supplementary Table S2, FD preprocessing markedly improved model performance, increasing $R_{p}^{2}$ to 0.8336 for SPXY+FD and 0.8423 for KS+FD. Figure 3H further illustrates that FD processing accentuated differences in absorption bands and shoulders by inducing oscillations around the baseline.

In the low-moisture system, overall spectral absorbance was substantially reduced, and water mainly existed in the bound form, resulting in highly overlapped spectral features between total water and bound water. Bound water tightly associated with solid matrices such as proteins and starch exhibited weak but positionally stable absorption peaks, without causing pronounced baseline drift.

For the low-moisture total water model, both smoothing and scatter correction improved model performance, with the SPXY+SG combination yielding the best result ($R_{p}^{2}$ = 0.9519). Figure 4C confirms that SG preprocessing effectively suppressed high-frequency noise. In the low-moisture bound water model, optimal performance was achieved using raw spectra combined with SPXY partitioning ($R_{p}^{2}$ = 0.9492), whereas excessive smoothing or derivative treatments tended to attenuate critical weak spectral features.

Under low-moisture conditions, the proportion of free water was extremely low, resulting in very weak absorption signals that were easily masked or distorted by noise, scattering, and baseline drift. Moreover, absorption from other sample constituents further obscured free water signals, preventing the extraction of stable spectral features. Consequently, the $R_{p}^{2}$ values of the low-moisture free water model remained at approximately 0.05, indicating insufficient data support for effective modeling; this model was therefore excluded from subsequent analyses.

Overall, optimal spectral preprocessing strategies varied markedly among different moisture systems and water types. In the full-moisture system, free water was more sensitive to scattering and noise, while bound water features were prone to masking by adjacent strong absorptions, making scatter correction and derivative enhancement particularly effective. In contrast, under low-moisture conditions, free water could not be reliably modeled due to extremely weak signals, whereas bound water models still achieved high predictive accuracy owing to relatively stable absorption positions. These findings underscore the necessity of tailoring sample partitioning and preprocessing strategies to the target water type, moisture range, and data distribution in NIR-based moisture quantification.

Model performance analysis revealed that, in the full-moisture system, predictive accuracy followed the order total water > free water > bound water, whereas in the low-moisture system, the trend was total water ≈ bound water ≫ free water. The optimal models were identified as follows: full-moisture total water (KS+MSC, $R_{p}^{2}$ = 0.9883), full-moisture free water (KS+FD, $R_{p}^{2}$ = 0.9620), full-moisture bound water (KS+FD, $R_{p}^{2}$ = 0.8423), low-moisture total water (SPXY+SG, $R_{p}^{2}$ = 0.9519), and low-moisture bound water (SPXY, $R_{p}^{2}$ = 0.9492).

#### 3.3.3. Analysis of NIR Spectral Feature Extraction

Based on the optimal sample partitioning and spectral preprocessing strategies determined for each moisture model, feature extraction algorithms were further applied to identify the most moisture-sensitive variables within the 10,000–4000 cm^−1^ spectral range, aiming to elucidate the spectral response characteristics of different water states.

1. CARS Feature Band Analysis

**Figure 5:**
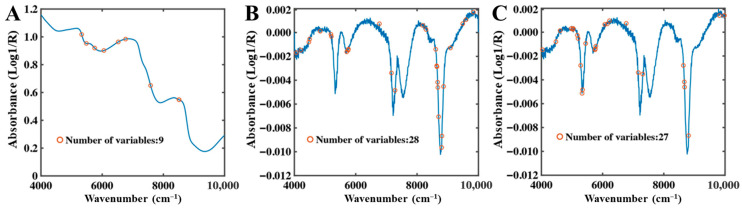
CARS selection results of full-moisture NIR spectra. (**A**) Total water, (**B**) Free water, (**C**) Bound water.

In the full-moisture system, only limited overlap was observed among the characteristic bands selected by CARS for total water, free water, and bound water (Figure 5). Since the total water signal represents the combined contribution of free and bound water, CARS preferentially retained key variables that best captured overall moisture variations, resulting in a relatively small number of selected bands. In contrast, partial overlap between the absorption features of free and bound water necessitated the selection of additional variables to discriminate their spectral characteristics.

**Figure 6:**
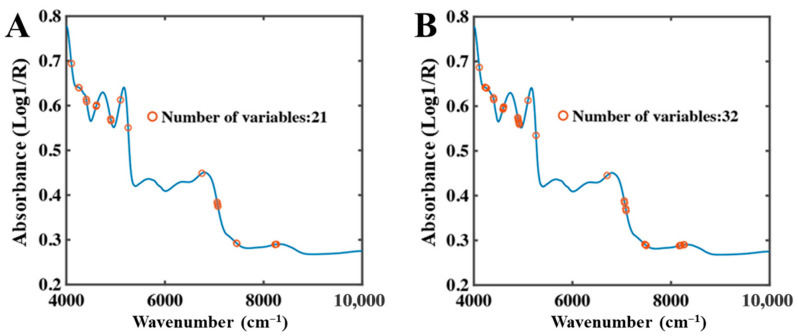
CARS selection results of low-moisture NIR spectra. (**A**) Total water, (**B**) Bound water.

In the low-moisture system, overall NIR absorbance decreased markedly, and spectral features were predominantly governed by bound water. Due to constraints imposed by the food matrix, bound water absorption peaks may shift or broaden, leading to more dispersed spectral responses. Consequently, CARS selected a larger number of bands to capture these complex features. As shown in Figure 6, total water and bound water shared eight characteristic bands, mainly located in the 5000–4000 cm^−1^ region.

2. SPA Feature Band Analysis

**Figure 7:**
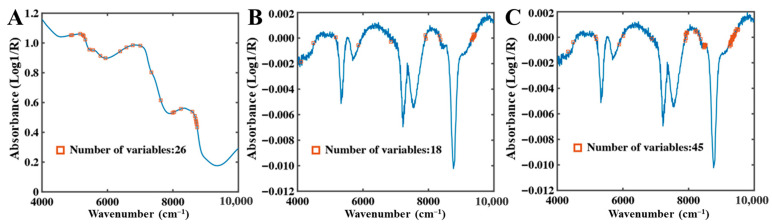
SPA selection results of full-moisture NIR spectra. (**A**) Total water, (**B**) Free water, (**C**) Bound water.

In the full-moisture system, SPA selected a total of 26 characteristic bands for total water, primarily distributed in the 9000–5000 cm^−1^ range (Figure 7), indicating pronounced NIR absorption features of total moisture within this region. Free water exhibited the fewest characteristic bands, reflecting its relatively concentrated absorption features affecting only specific wavelengths. In contrast, bound water displayed the largest number and widest distribution of characteristic bands, owing to pronounced matrix-binding effects; thus, SPA retained more variables to ensure model stability. Notably, bound water bands were densely distributed in the 9500–8200 cm^−1^ interval, suggesting strong absorption sensitivity in this region.

**Figure 8:**
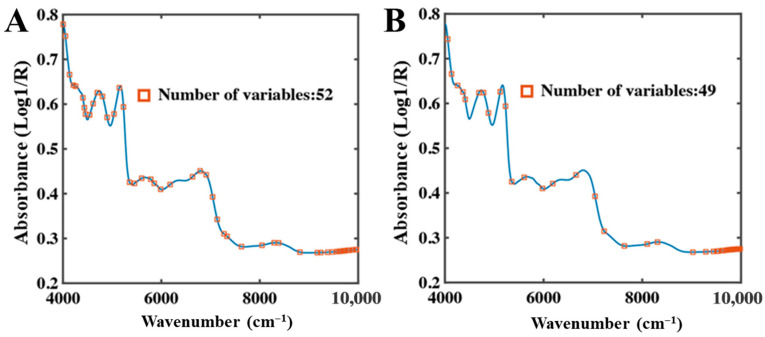
SPA selection results of low-moisture NIR spectra. (**A**) Total water, (**B**) Bound water.

In the low-moisture system, the number of SPA-selected variables increased substantially, with bands distributed across 9800–4000 cm^−1^ (Figure 8). This trend was consistent with the CARS results; however, unlike CARS, which retains only a limited number of representative variables, SPA preserves more spectral information while reducing dimensionality. This characteristic enhances model generalization under low-moisture conditions and enables more accurate representation of spectral variations in total and bound water.

3. UVE Feature Band Analysis

**Figure 9:**
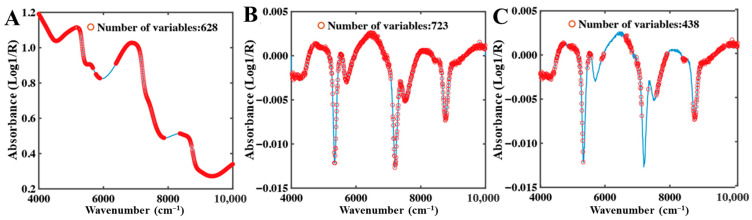
UVE selection results of full-moisture NIR spectra. (**A**) Total water, (**B**) Free water, (**C**) Bound water.

Compared with CARS and SPA, UVE selected a markedly larger number of variables (Figure 9). In the full-moisture system, free water dominated spectral variations, resulting in a high degree of overlap between the characteristic bands of total water and free water. Owing to the relatively loose molecular state of free water and its complex spectral responses, UVE retained more variables to comprehensively describe its features. In contrast, bound water, which is tightly associated with the food matrix, exhibited more stable absorption peaks, leading to fewer selected bands.

**Figure 10:**
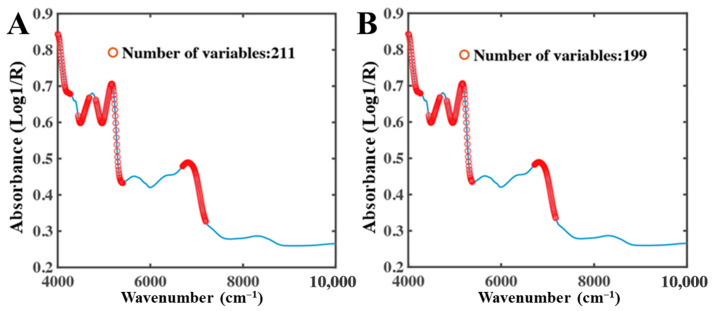
UVE selection results of low-moisture NIR spectra. (**A**) Total water, (**B**) Bound water.

Under low-moisture conditions, the number of UVE-selected variables decreased relative to the full-moisture system (Figure 10). The characteristic bands of total water and bound water remained highly consistent, mainly distributed in the 4300–4000 cm^−1^, 5400–4440 cm^−1^, and 5400–4800 cm^−1^ regions. These bands were also retained in the full-moisture system, indicating their robust capability to characterize moisture information across different moisture levels.

Overall, CARS, SPA, and UVE exhibited distinct performances in terms of dimensionality reduction and feature band distribution. CARS provided the strongest variable reduction but may risk information loss; SPA achieved a favorable balance between dimensionality reduction and information retention, yielding concentrated and less redundant feature sets that enhance model stability; UVE exhibited the weakest reduction ability but preserved more comprehensive spectral information, making it suitable for moisture systems with complex spectral responses.

### 3.4. Performance Analysis of Near-Infrared Based Prediction Models for Total Moisture, Bound Water and Free Water

The moisture content prediction models were established using the characteristic wavelength subsets screened in Section 3.3.3. The applicability of NIR spectroscopy in both full-moisture and low-moisture systems was systematically investigated, and its predictive performance for total water, free water and bound water was evaluated.

Supplementary Tables S4 and S5 present a comparative analysis of the predictive performance of five machine learning methods. SVR and Back Propagation Artificial Neural Network (BPANN) exhibited more robust performance across moisture systems. SVR, possesses strong nonlinear capability and noise resistance via kernel methods, while BPANN effectively learns complex features through multi-layer neural networks. In contrast, linear PLSR yielded lower prediction accuracy. XGBoost performed excellently in the training set ($R_{p}^{2}$ > 0.99) but suffered from overfitting in the test set. Zhang et al. [24] attributed this phenomenon to the reduction in model input variables after feature extraction, which led to insufficient learning of the decision tree structure and failure to effectively construct multi-layer decision paths. The performance of the PSO-RF model was generally inferior, probably due to inaccurate feature selection, which prevented the random forest from fully utilizing effective information during the learning process.

Feature extraction methods exerted a significant impact on the predictive performance of the models. Overall, CARS achieved the highest prediction accuracy in most models. The Successive Projections Algorithm (SPA) performed slightly worse than CARS in some cases, while UVE retained the most variables but yielded poorer predictive performance.

Remarkable differences existed in the prediction accuracy of various water types under different moisture systems. In the full-moisture system, the prediction accuracy of total water and free water was the highest, with $R_{p}^{2}$ values of 0.9902 and 0.9740, respectively. Nevertheless, the prediction accuracy of bound water was relatively low ($R_{p}^{2}$ = 0.8911), which might be ascribed to the weakened spectral contribution of bound water by the strong signal of free water. In the low-moisture system, free water became difficult to predict, and the prediction accuracy of total water decreased ($R_{p}^{2}$ = 0.9646), suggesting that the weakened spectral signal with reduced moisture content impeded the model from learning subtle moisture variations. Conversely, the prediction accuracy of bound water was improved ($R_{p}^{2}$ = 0.9709), owing to the more stable and prominent spectral signal of bound water in the low-moisture environment.

**Figure 11:**
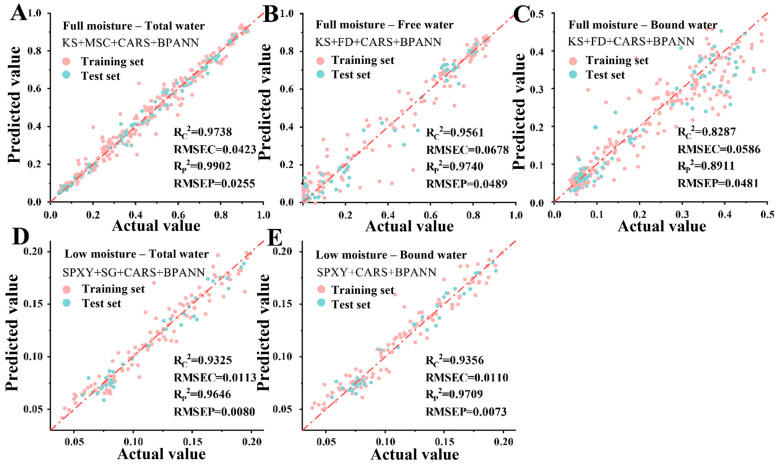
Scatter plot of the optimal moisture content prediction model based on NIR spectra. (**A**) Full-moisture total water model, (**B**) full-moisture free water model, (**C**) full-moisture bound water model, (**D**) low-moisture total water model, (**E**) low-moisture bound water model; R^2^: coefficient of determination, RMSE: root mean square error.

In summary, the optimal combinations of sample classification, preprocessing, feature extraction and modeling methods for different water types based on NIR spectroscopy varied with application scenarios. Figure 11 displays the scatter plots of measured versus predicted values for the five optimal moisture prediction models. Among them, the full-moisture total water model (Figure 11A) achieved the best performance, with both training and test set samples highly concentrated around the regression line, indicating superior prediction accuracy and generalization ability. However, the scatter distribution of the full-moisture bound water model (Figure 11C) showed a certain deviation, consistent with its lower R^2^ and higher Root Mean Square Error (RMSE), which verified the limited modeling accuracy and greater prediction difficulty of bound water under full-moisture conditions. On the whole, the sample points of all models were favorably distributed near the Y = X regression line, demonstrating that the established models possessed satisfactory fitting performance and high robustness, and were suitable for the rapid quantitative prediction of moisture content during carrot freeze-drying.

## 4. Conclusions

This study validated the feasibility of near-infrared (NIR) spectroscopy (10,000–4000 cm^−1^) for rapid, non-destructive detection of total, free, and bound water in carrot slices during freeze-drying, coupled with LF-NMR for water state characterization and machine learning for quantitative modeling. Optimized spectral processing strategies were water-type- and moisture-system-specific: MSC/FD preprocessing performed best for full-moisture samples, SG smoothing for low-moisture total water, and CARS was the optimal feature extraction method. Among five machine learning models, SVR and BPANN exhibited robust nonlinear modeling and adaptability to diverse moisture systems, outperforming PLSR, XGBoost (prone to overfitting), and PSO-RF. The full-moisture NIR model achieved high prediction accuracy for total water ($R_{p}^{2}$ = 0.9902) and free water ($R_{p}^{2}$ = 0.9740), while bound water prediction was limited by free water signal masking; in the low-moisture system, bound water prediction accuracy improved significantly ($R_{p}^{2}$ = 0.9709), and free water could not be effectively modeled due to ultra-weak signals. All optimal models presented excellent fitting and generalization ability, confirming NIR spectroscopy as a reliable tool for rapid moisture quantification in carrot freeze-drying. This research provides a technical reference for non-destructive monitoring of fruit and vegetable freeze-drying. Comparisons with studies specifically conducted on carrot slices further contextualize the performance of our NIR-based models. Long et al. [25] reported predicting moisture content during hot-air drying of carrot slices using multispectral imaging coupled with SVM models, achieving high predictive performance (R^2^ ≈ 0.99). Similarly, Zhou et al. [26] developed a C-LSTM model based on multispectral imaging for moisture prediction in carrot slices during drying, reporting high predictive accuracy (R_p_ ≈ 0.96). Although these studies employed different spectroscopic approaches (multispectral imaging versus NIR spectroscopy), their findings are consistent with our results, demonstrating that spectroscopic techniques combined with machine learning can achieve excellent predictive accuracy for moisture content in carrot slices. Notably, the present study extends these approaches by differentiating between free water and bound water and by developing stage-specific models for full-moisture and low-moisture systems, which has not been previously reported for carrot freeze-drying. Future work will focus on portable NIR-based online real-time monitoring to enhance industrial applicability, optimize model transferability for different fruits and vegetables, and integrate deep learning to improve bound water prediction accuracy in high-moisture systems. Additionally, combining NIR and LF-NMR technologies to construct a comprehensive moisture detection system will be explored for intelligent control of agricultural product freeze-drying processes.

## Figures and Tables

**Table 1:** Functional groups and vibrational modes of water involved in NIR spectroscopy.

Functional Groups	Region	Wavenumber (cm^−1^)	Wavelength (nm)	Vibration Distribution	Physical Significance
O-H (H_2_O)	2	8220–7015	1216–1425	First harmonic	Mainly reflects the first overtone vibration characteristics of the O-H bond in water molecules.
	3	6800–5785	1470–1728	Combined frequency	Related to the vibration mode within water molecules, it is applicable for water analysis.
	4	5781–5226	1730–1913	Combined frequency	Reflecting the coupling of different O-H vibration modes, it is suitable for supplementary quantitative analysis of water content.

**Table 2:** Vacuum freeze-drying parameters.

Stage	Temperature/°C	Time/h
1	−35	4
2	−30	1
3	−20	1
4	−10	5
5	0	3
6	10	6
7	20	4
8	30	3
9	45	5

**Table 3:** Descriptive statistics of carrot moisture changes during the freeze-drying process.

Moisture Gradient (%)	A21	A22	A23	A24	Bound Water (%)	Free Water (%)	Total Water (%)
90–100	93.48 ± 16.06	91.85 ± 25.62	152.94 ± 35.06	4598.39 ± 124.14	5.52 ± 0.52	85.99 ± 0.72	91.51 ± 0.89
80–90	39.67 ± 19.84	69.58 ± 21.66	102.27 ± 24.52	2758.32 ± 582.58	5.67 ± 1.15	79.57 ± 3.17	85.24 ± 2.29
70–80	56.00 ± 30.22	59.70 ± 26.89	80.99 ± 28.53	1631.50 ± 384.56	7.64 ± 2.41	67.28 ± 3.67	74.92 ± 2.79
60–70	63.58 ± 32.23	79.88 ± 31.07	237.35 ± 151.81	583.56 ± 284.81	25.90 ± 12.80	39.11 ± 12.88	65.01 ± 2.99
50–60	64.94 ± 27.67	103.62 ± 31.11	372.46 ± 164.51	291.40 ± 235.67	36.41 ± 10.53	18.80 ± 11.31	55.21 ± 2.64
40–50	85.27 ± 49.53	109.06 ± 42.23	399.88 ± 150.45	59.18 ± 62.58	40.82 ± 4.52	4.25 ± 4.34	45.06 ± 2.99
30–40	92.99 ± 46.29	139.52 ± 60.70	330.52 ± 140.94	15.37 ± 17.74	34.36 ± 2.74	1.04 ± 1.23	35.40 ± 2.63
20–30	150.61 ± 78.10	200.26 ± 81.05	124.95 ± 105.70	7.32 ± 5.79	24.95 ± 2.75	0.39 ± 0.34	25.34 ± 2.73
10–20	163.95 ± 63.13	105.10 ± 77.28	21.62 ± 14.55	5.00 ± 1.98	14.84 ± 2.95	0.29 ± 0.16	15.13 ± 2.94
0–10	88.14 ± 49.24	55.94 ± 18.14	16.94 ± 6.70	4.88 ± 2.42	6.63 ± 1.69	0.20 ± 0.10	6.83 ± 1.70

## Data Availability

The original contributions presented in this study are included in the article/Supplementary Materials. Further inquiries can be directed to the corresponding author.

## Supplementary Materials

The following supporting information can be downloaded at https://www.mdpi.com/article/10.3390/foods15071256/s1, Table S1: Descriptive statistics of the dataset under different sample partitioning methods; Table S2: Summary of full moisture quantitative prediction models using different preprocessing methods; Table S3: Summary of low moisture quantitative prediction models using different preprocessing methods; Table S4: Summary of full-moisture quantitative prediction models; Table S5: Summary of low-moisture quantitative prediction models.

## References

[1] Squeo G., Cruz J., De Angelis D., Caponio F., Amigo J. (2024). Considerations about the gap between research in near-infrared spectroscopy and official methods and recommendations of analysis in foods. Curr. Opin. Food Sci..

[2] Li J., Li Z., Wang N., Raghavan G.S.V., Pei Y., Song C., Zhu G. (2020). Novel Sensing Technologies During the Food Drying Process. Food Eng. Rev..

[3] Fodor M., Matkovits A., Benes E., Jókai Z. (2024). The Role of Near-Infrared Spectroscopy in Food Quality Assurance: A Review of the Past Two Decades. Foods.

[4] Zhang M., Lin B., Zhang S., Peng C., Li C., Feng T., Li L., Wu A., Yang C., Wang W. (2025). Application of artificial intelligence in the rapid determination of moisture content in medicine food homology substances. Food Chem..

[5] Massei A., Falco N., Fissore D. (2023). Use of machine learning tools and NIR spectra to estimate residual moisture in freeze-dried products. Spectrochim. Acta Part A Mol. Biomol. Spectrosc..

[6] Jin W., Zhang M., Mujumdar A., Yu D. (2024). Application of Portable NIR Spectroscopy for Instant Prediction of the Product Quality of Apple Slices During Hot Air-Assisted Radio Frequency Drying. Food Bioprocess Technol..

[7] Marinoni L., Cattaneo T., Vanoli M., Barzaghi S. (2023). Real-time monitoring of solar drying of melon slices with a portable NIR spectrometer: A preliminary approach. Eur. Food Res. Technol..

[8] Zhang W., Pan M., Wang P., Xue J., Zhou X., Sun W., Hu Y., Shen Z. (2024). Comparative Analysis of XGB, CNN, and ResNet Models for Predicting Moisture Content in *Porphyra yezoensis* Using Near-Infrared Spectroscopy. Foods.

[9] Zou X., Wang Q., Chen Y., Wang J., Xu S., Zhu Z., Yan C., Shan P., Wang S., Fu Y. (2025). Fusion of convolutional neural network with XGBoost feature extraction for predicting multi-constituents in corn using near infrared spectroscopy. Food Chem..

[10] Purwanto Y.A., Widodo S., Iriani E.S. (2024). Rapid assessment of vanilla (*Vanilla planifolia*) quality parameters using portable near-infrared spectroscopy combined with random forest. J. Food Compos. Anal..

[11] Parrenin L., Danjou C., Agard B., Marchesini G., Barbosa F. (2024). A decision support tool to analyze the properties of wheat, cocoa beans and mangoes from their NIR spectra. J. Food Sci..

[12] Ordoñez-Lozano S., Collazos-Escobar G., Bahamón-Monje A., Gutiérrez-Guzmán N. (2025). Monitoring moisture content in parchment coffee beans during drying using Fourier Transform near infrared (FT-NIR) spectroscopy: A dataset for calibrating chemometric-based models for moisture prediction. Data Brief.

[13] (2021). Freeze-Dried Fruits and Vegetables.

[14] (2016). National Food Safety Standard—Determination of Moisture in Foods.

[15] Sun Y., Zhang M., Mujumdar A., Yu D. (2021). Pulse-spouted microwave freeze drying of raspberry: Control of moisture using ANN model aided by LF-NMR. J. Food Eng..

[16] Li Y., Gao H., Wan Q., Qiu X., Qi Y., Wu Z. (2024). Investigation of water variation and surface crust of *Polygonati rhizoma* extract during vacuum drying. LWT-Food Sci. Technol..

[17] Chen H., Shen Y. (2023). Investigation of Water Distribution and Mobility Dynamics in Recalcitrant *Quercus acutissima* Seeds during Desiccation Using Magnetic Resonance Methods. Forests.

[18] Song Y., Yi W., Liu Y., Zhang C., Wang Y., Ning J. (2025). A robust deep learning model for predicting green tea moisture content during fixation using near-infrared spectroscopy: Integration of multi-scale feature fusion and attention mechanisms. Food Res. Int..

[19] Yue J., Zhang H., Gao L., Tian W., Luo J., Nie L., Li L., Wu A., Zang H. (2025). Benchtop and different miniaturized near-infrared spectrometers application study: Calibration transfer and 2D-COS for in-situ analysis of moisture content in HPMC. Spectrochim. Acta Part-Mol. Biomol. Spectrosc..

[20] Zang Z., Li Z., Lu X., Liang J., Wang J., Cui H., Yan S. (2021). Terahertz spectroscopy for quantification of free water and bound water in leaf. Comput. Electron. Agric..

[21] Wang R., Song J., Liu J., Ren Z., Zhu C., Yu Y., Li Z., Huang Y. (2024). The combination of near-infrared spectroscopy with chemometrics in achieving rapid and accurate determination of rice mildew. J. Food Meas. Charact..

[22] Wei W., Zhang F., Fu F., Sang S., Qiao Z. (2023). Rapid Detection of Total Viable Count in Intact Beef Dishes Based on NIR Hyperspectral Hybrid Model. Sensors.

[23] Liu Y., Zhou X., Sun J., Li B., Ji J. (2024). A Method for Non-destructive Detection of Moisture Content in Oilseed Rape Leaves Using Hyperspectral Imaging Technology. J. Nondestruct. Eval..

[24] Zhang R., Jiang L., Duan N., Fu W., Ma J., Sun X., Liao J., Jin H. (2024). High-accuracy quantitative model for phosphate anions in solution based on absorption spectroscopy and machine learning algorithms. J. Clean. Prod..

[25] Long J., Yang J., Peng J., Pan L., Tu K. (2021). Detection of moisture and carotenoid content in carrot slices during hot air drying based on multispectral imaging equipment with selected wavelengths. Int. J. Food Eng..

[26] Zhou H., Huang M., Zhu Q., Zhang M. (2022). Developing C-LSTM model for evaluating moisture content of carrot slices during drying. Dry. Technol..

