# Supplementary material for: Application of Near-Infrared Spectroscopy in Moisture Detection of Carrot Slices During Freeze-Drying
Source: Foods. 2026 Apr 7;15(7):1256. doi: 10.3390/foods15071256 (PMC13073678; doi:10.3390/foods15071256)
Supplement: Supplementary file 1 [file foods-15-01256-s001.zip › foods-4218348-supplementary.pdf]

Table S1. Descriptive statistics of the dataset under different sample partitioning methods

Water content range	Sample division	Type of water content	Dataset	Quantity	Minimum value	Maximum value	Average value	Standard deviation
Total Moisture MC<100%	KS	Total water	Training set	225	0.0422	0.9272	0.4855	0.2624
			Test set	75	0.0400	0.9173	0.4663	0.2595
		Free water	Training set	225	0.0000	0.8739	0.2671	0.3246
			Test set	75	0.0002	0.8559	0.2528	0.3050
		Bound water	Training set	225	0.0377	0.5738	0.2184	0.1418
			Test set	75	0.0385	0.4680	0.2135	0.1468
	SPXY	Total water	Training set	225	0.0400	0.9272	0.4822	0.2617
			Test set	75	0.0566	0.9173	0.4763	0.2623
		Free water	Training set	225	0.0000	0.8739	0.2640	0.3212
			Test set	75	0.0002	0.8559	0.2619	0.3160
		Bound water	Training set	225	0.0385	0.5738	0.2181	0.1434
			Test set	75	0.0377	0.4871	0.2144	0.1422
Low Moisture MC<20%	KS	Total water	Training set	105	0.0400	0.1992	0.1176	0.0425
			Test set	35	0.0422	0.1937	0.1146	0.0463
		Free water	Training set	105	0.0000	0.0086	0.0026	0.0020
			Test set	35	0.0000	0.0084	0.0024	0.0019
		Bound water	Training set	105	0.0385	0.1992	0.1149	0.0423
			Test set	35	0.0403	0.1937	0.1122	0.0466
	SPXY	Total water	Training set	105	0.0400	0.1992	0.1174	0.0436
			Test set	35	0.0574	0.1937	0.1152	0.0431
		Free water	Training set	105	0.0000	0.0086	0.0026	0.0020
			Test set	35	0.0000	0.0084	0.0025	0.0020
		Bound water	Training set	105	0.0385	0.1992	0.1148	0.0434
			Test set	35	0.0550	0.1937	0.1127	0.0435

Table S2. Summary of full moisture quantitative prediction models using different preprocessing methods

**Sample division**	**Pre-treatment**	**Type of water content**											
		**Total water**				**Free water**				**Bound water**			
		R_C^2	***RMSEC***	***R_P^2***	***RMSEP***	***R_C^2***	***RMSEC***	***R_P^2***	***RMSEP***	***R_C^2***	***RMSEC***	***R_P^2***	***RMSEP***
**KS**	/	0.9741	0.0422	0.9860	0.0312	0.9399	0.0796	0.9563	0.0682	0.7756	0.0671	0.8176	0.0627
	MA	0.9741	0.0421	0.9860	0.0312	0.9399	0.0796	0.9562	0.0683	0.7754	0.0672	0.8172	0.0628
	SG	0.9742	0.0421	0.9859	0.0312	0.9399	0.0796	0.9563	0.0682	0.7756	0.0671	0.8176	0.0627
	SNV	0.9724	0.0435	0.9880	0.0290	0.9353	0.0826	0.9506	0.0685	0.7285	0.0739	0.7635	0.0712
	Nor	0.9710	0.0447	0.9858	0.0314	0.9360	0.0820	0.9520	0.0675	0.7973	0.0639	0.8199	0.0619
	MSC	**0.9732**	**0.0429**	**0.9883**	**0.0282**	0.9241	0.0894	0.9396	0.0762	0.7735	0.0674	0.8122	0.0632
	OSC	0.9289	0.0702	0.9399	0.0642	0.8190	0.1340	0.8900	0.1023	0.7898	0.0653	0.8064	0.0670
	FD	0.9749	0.0415	0.9851	0.0320	**0.9569**	**0.0673**	**0.9620**	**0.0600**	**0.8136**	**0.0611**	**0.8423**	**0.0580**
	SD	0.9967	0.0152	0.9755	0.0413	0.9539	0.0696	0.9467	0.0717	0.8519	0.0545	0.8075	0.0646
**SPXY**	/	0.9726	0.0433	0.9870	0.0304	0.9483	0.0732	0.9537	0.0700	0.7958	0.0647	0.8087	0.0620
	MA	0.9726	0.0433	0.9870	0.0304	0.9483	0.0732	0.9537	0.0699	0.7954	0.0648	0.8090	0.0619
	SG	0.9726	0.0433	0.9870	0.0303	0.9483	0.0732	0.9537	0.0700	0.7958	0.0647	0.8087	0.0619
	SNV	0.9670	0.0475	0.9869	0.0298	0.9373	0.0722	0.9373	0.0804	0.7549	0.0709	0.7971	0.0636
	Nor	0.9709	0.0446	0.9868	0.0301	0.9310	0.0844	0.9460	0.0730	0.8106	0.0623	0.7940	0.0647
	MSC	0.9668	0.0476	0.9875	0.0290	0.9370	0.0806	0.9473	0.0721	0.7527	0.0712	0.7996	0.0633
	OSC	0.9047	0.0809	0.9083	0.0797	0.7997	0.1472	0.8422	0.1297	0.7808	0.0672	0.7701	0.0690
	FD	0.9740	0.0421	0.9874	0.0297	0.9678	0.0577	0.9578	0.0648	0.8200	0.0607	0.8336	0.0577
	SD	0.9966	0.0154	0.9722	0.0455	0.9752	0.0507	0.9493	0.0728	0.8726	0.0511	0.8259	0.0590

Table S3. Summary of low moisture quantitative prediction models using different preprocessing methods

Sample division	Pretreatment	Type of water content											
		Total water				Free water				Bound water			
		R_C^2	*RMSEC*	R_P^2	*RMSEP*	R_C^2	*RMSEC*	R_P^2	*RMSEP*	R_C^2	*RMSEC*	R_P^2	*RMSEP*
**KS**	/	0.9194	0.0120	0.9260	0.0134	0.4938	0.0015	0.0111	0.0020	0.9314	0.0110	0.9293	0.0132
	MA	0.9187	0.0121	0.9259	0.0134	0.4932	0.0015	0.0111	0.0020	0.9299	0.0112	0.9292	0.0131
	SG	0.9192	0.0120	0.9263	0.0134	0.4936	0.0015	0.0109	0.0020	0.9308	0.0111	0.9294	0.0131
	SNV	0.9364	0.0107	0.9485	0.0112	0.2422	0.0018	0.0148	0.0019	0.9149	0.0124	0.9240	0.0143
	Nor	0.9554	0.0089	0.9440	0.0120	0.1366	0.0020	0.0581	0.0018	0.9584	0.0086	0.9411	0.0123
	MSC	0.9360	0.0107	0.9480	0.0112	0.2420	0.0018	0.0117	0.0019	0.9154	0.0123	0.9249	0.0142
	OSC	0.6129	0.0268	0.3509	0.0388	0.7249	0.0018	0.0000	0.0019	0.8475	0.0167	0.4955	0.0335
	FD	0.9259	0.0115	0.9194	0.0138	0.4465	0.0016	0.0211	0.0019	0.9447	0.0099	0.9219	0.0136
	SD	0.9944	0.0034	0.9125	0.0156	0.7334	0.0013	0.0521	0.0018	0.9957	0.0030	0.9121	0.0159
**SPXY**	/	0.9461	0.0101	0.9518	0.0102	0.4421	0.0015	0.0753	0.0019	0.9390	0.0107	0.9492	0.0104
	MA	0.9455	0.0102	0.9518	0.0102	0.4414	0.0015	0.0751	0.0019	0.9381	0.0107	0.9487	0.0105
	SG	0.9458	0.0101	0.9519	0.0101	0.4419	0.0015	0.0753	0.0019	0.9386	0.0107	0.9490	0.0104
	SNV	0.9236	0.0121	0.9299	0.0115	0.4675	0.0015	0.0159	0.0020	0.9302	0.0115	0.9329	0.0113
	Nor	0.9415	0.0105	0.9372	0.0119	0.7726	0.0011	0.0396	0.0019	0.9230	0.0120	0.9146	0.0144
	MSC	0.9240	0.0120	0.9330	0.0112	0.5087	0.0015	0.0024	0.0021	0.9299	0.0115	0.9347	0.0111
	OSC	0.8288	0.0182	0.2794	0.0406	0.1248	0.0019	0.0027	0.0020	0.8278	0.0182	0.2564	0.0418
	FD	0.9815	0.0059	0.9470	0.0109	0.4646	0.0016	0.0250	0.0020	0.9915	0.0040	0.9426	0.0114
	SD	0.9988	0.0016	0.8991	0.0142	0.7127	0.0014	0.0249	0.0019	0.9989	0.0016	0.9024	0.0141

Table S4. Summary of full-moisture quantitative prediction models

**Models**	**Feature extraction**		**Type of water content**													
			**Total water**					**Free water**					**Bound water**			
		R_C^2	*RMSEC*	R_P^2	*RMSEP*	R_C^2	*RMSEC*	R_P^2	*RMSEP*	R_C^2	*RMSEC*	R_P^2	*RMSEP*	R_C^2	*RMSEC*	R_P^2
**PLSR**	/	778	0.9620	0.0501	0.9817	0.0340	778	0.9078	0.0941	0.9195	0.0796	778	0.6592	0.0714	0.7289	0.0682
	CARS	9	0.9693	0.0452	0.9851	0.0312	28	0.9430	0.0752	0.9498	0.0648	27	0.7983	0.058	0.8011	0.0597
	SPA	26	0.9739	0.0418	0.9864	0.0298	18	0.9156	0.0904	0.9399	0.0716	45	0.7815	0.0599	0.8045	0.0605
	UVE	628	0.9818	0.0350	0.9761	0.0399	723	0.978	0.0454	0.9126	0.0887	438	0.6499	0.0721	0.7194	0.0689
**SVR**	/	778	0.9732	0.0429	0.9883	0.0282	778	0.9569	0.0673	0.9620	0.0600	778	0.8136	0.0611	0.8423	0.0580
	CARS	9	0.9726	0.0434	0.9892	0.0270	28	0.9503	0.0723	0.9685	0.0542	27	0.8296	0.0585	0.8561	0.0561
	SPA	26	0.9732	0.0429	0.9888	0.0278	18	0.9349	0.0827	0.9500	0.0686	45	0.8103	0.0617	0.8228	0.0616
	UVE	628	0.9736	0.0426	0.9882	0.0286	723	0.9558	0.0682	0.9617	0.0603	438	0.8120	0.0614	0.8322	0.0602
**BPANN**	/	778	0.9805	0.0366	0.9886	0.0275	778	0.9570	0.0672	0.9611	0.0597	778	0.8533	0.0542	0.8445	0.0575
	CARS	**9**	**0.9738**	**0.0423**	**0.9902**	**0.0255**	28	**0.9561**	**0.0678**	**0.9740**	**0.0489**	27	**0.8287**	**0.0586**	**0.8911**	**0.0481**
	SPA	26	0.9757	0.0408	0.9896	0.0263	18	0.9501	0.0723	0.9601	0.0606	45	0.8363	0.0573	0.8372	0.0588
	UVE	628	0.9791	0.0379	0.9882	0.0280	723	0.9592	0.0654	0.9487	0.0686	438	0.8287	0.0586	0.8442	0.0576
**XGBoost**	/	778	0.9999	0.0014	0.9868	0.0296	778	0.9999	0.0007	0.9652	0.0566	778	0.9999	0.0015	0.8513	0.0562
	CARS	9	0.9995	0.0057	0.9794	0.0370	28	0.9999	0.0025	0.9703	0.0522	27	0.9984	0.0057	0.8569	0.0552
	SPA	26	0.9999	0.0032	0.9842	0.0325	18	0.9999	0.0034	0.9423	0.0728	45	0.9996	0.0028	0.8220	0.0615
	UVE	628	0.9999	0.0018	0.9869	0.0295	723	0.9999	0.0008	0.9578	0.0622	438	0.9999	0.0013	0.8374	0.0588
**PSO-RF**	/	778	0.9936	0.0209	0.9872	0.0291	778	0.9873	0.0365	0.9619	0.0592	778	0.9551	0.0300	0.8732	0.0519
	CARS	9	0.9922	0.0232	0.9863	0.0302	28	0.9862	0.0380	0.9688	0.0535	27	0.9481	0.0322	0.8724	0.0521
	SPA	26	0.9930	0.0219	0.9874	0.0289	18	0.9814	0.0442	0.9642	0.0573	45	0.9508	0.0313	0.8410	0.0582
	UVE	628	0.9934	0.0213	0.9883	0.0279	723	0.9880	0.0355	0.9574	0.0625	438	0.9571	0.0293	0.8792	0.0507

Table S5. Summary of low-moisture quantitative prediction models

**Models**	**Feature extraction**	**Type of water content**									
		**Total water**					**Bound water**				
		**variable**	***Rc*²**	***RMSEC***	***Rc*²**	***RMSEC***	**variable**	***Rc*²**	***RMSEC***	***Rc*²**	***RMSEC***
**PLSR**	/	778	0.6744	0.0215	0.7916	0.0179	778	0.6841	0.0212	0.7919	0.0180
	CARS	21	0.9116	0.0124	0.9122	0.0125	32	0.9247	0.0114	0.8657	0.0148
	SPA	52	0.9747	0.0068	0.9551	0.0088	49	0.9623	0.0082	0.9290	0.0115
	UVE	211	0.6993	0.0208	0.7692	0.0177	199	0.7037	0.0206	0.7695	0.0178
**SVR**	/	778	0.9458	0.0101	0.9519	0.0101	778	0.9390	0.0107	0.9492	0.0104
	CARS	21	0.9278	0.0116	0.9389	0.0116	32	0.9202	0.0123	0.9310	0.0119
	SPA	52	0.9408	0.0106	0.9526	0.0099	49	0.9252	0.0118	0.9253	0.0129
	UVE	211	0.9058	0.0134	0.9323	0.0113	199	0.9042	0.0135	0.9163	0.0128
**BPANN**	/	778	0.9145	0.0127	0.9491	0.0096	778	0.9148	0.0126	0.9359	0.0109
	CARS	21	**0.9325**	**0.0113**	**0.9646**	**0.0080**	32	**0.9356**	**0.0110**	**0.9709**	**0.0073**
	SPA	52	0.9097	0.013	0.9623	0.0083	49	0.8996	0.0137	0.9456	0.0100
	UVE	211	0.8867	0.0146	0.9404	0.0104	199	0.9104	0.0129	0.9310	0.0113
**XGBoost**	/	778	0.9999	0.0004	0.8467	0.0166	778	0.9999	0.0004	0.8888	0.0143
	CARS	21	0.9997	0.0008	0.8474	0.0166	32	0.9996	0.0008	0.8359	0.0174
	SPA	52	0.9999	0.0005	0.8721	0.0152	49	0.9998	0.0006	0.8773	0.0150
	UVE	211	0.9997	0.0007	0.8136	0.0184	199	0.9998	0.0007	0.7824	0.0200
**PSO-RF**	/	778	0.9390	0.0107	0.8806	0.0147	778	0.9393	0.0106	0.8729	0.0153
	CARS	21	0.9432	0.0103	0.8713	0.0152	32	0.9387	0.0107	0.8652	0.0157
	SPA	52	0.9339	0.0112	0.8862	0.0143	49	0.9365	0.0109	0.8723	0.0153
	UVE	211	0.9402	0.0105	0.8421	0.0169	199	0.9381	0.0107	0.8316	0.0176
